# DCE-MRI for Pre-Treatment Prediction and Post-Treatment Assessment of Treatment Response in Sites of Squamous Cell Carcinoma in the Head and Neck

**DOI:** 10.1371/journal.pone.0144770

**Published:** 2015-12-10

**Authors:** Ann D. King, Steven Kwok Keung Chow, Kwok-Hung Yu, Frankie Kwok Fai Mo, David K. W. Yeung, Jing Yuan, Benjamin King Hong Law, Kunwar S. Bhatia, Alexander C. Vlantis, Anil T. Ahuja

**Affiliations:** 1 Department of Imaging and Interventional Radiology, The Chinese University of Hong Kong, Prince of Wales Hospital, Hong Kong S.A.R., China; 2 School of Physics, Faculty of Engineering and Information Sciences, University of Wollongong, Australia; 3 Alpha Oncology Centre, Hong Kong S.A.R., China; 4 Department of Clinical Oncology, The Chinese University of Hong Kong, Prince of Wales Hospital, Hong Kong S.A.R., China; 5 Medical Physics and Research Department, Hong Kong Sanatorium & Hospital, Hong Kong S.A.R., China; 6 Department of Otorhinolaryngology, Head and Neck Surgery, The Chinese University of Hong Kong, Prince of Wales Hospital, Hong Kong S.A.R. China; University of Cincinnati College of Medicine, UNITED STATES

## Abstract

**Background and Purpose:**

It is important to identify patients with head and neck squamous cell carcinoma (SCC) who fail to respond to chemoradiotherapy so that they can undergo post-treatment salvage surgery while the disease is still operable. This study aimed to determine the diagnostic performance of dynamic contrast enhanced (DCE)-MRI using a pharmacokinetic model for pre-treatment predictive imaging, as well as post-treatment diagnosis, of residual SCC at primary and nodal sites in the head and neck.

**Material and Methods:**

Forty-nine patients with 83 SCC sites (primary and/or nodal) underwent pre-treatment DCE-MRI, and 43 patients underwent post-treatment DCE-MRI, of which 33 SCC sites had a residual mass amenable to analysis. Pre-treatment, post-treatment and % change in the mean K^trans^, k_ep_, v_e_ and AUGC were obtained from SCC sites. Logistic regression was used to correlate DCE parameters at each SCC site with treatment response at the same site, based on clinical outcome at that site at a minimum of two years.

**Results:**

None of the pre-treatment DCE-MRI parameters showed significant correlations with SCC site failure (SF) (29/83 sites) or site control (SC) (54/83 sites). Post-treatment residual masses with SF (14/33) had significantly higher k_ep_ (p = 0.05), higher AUGC (p = 0.02), and lower % reduction in AUGC (p = 0.02), than residual masses with SC (19/33), with the % change in AUGC remaining significant on multivariate analysis.

**Conclusion:**

Pre-treatment DCE-MRI did not predict which SCC sites would fail treatment, but post-treatment DCE-MRI showed potential for identifying residual masses that had failed treatment.

## Introduction

While neovascularization is necessary for the growth and spread of head and neck squamous cell carcinomas (SCC), it also influences the response to chemoradiotherapy (CRT) and radiotherapy (RT). Dynamic contrast enhanced MRI (DCE-MRI) provides a non-invasive *in-vivo* method to evaluate the perfusion and permeability of these abnormal blood vessels and so provides an indirect method of assessing the hypoxic nature of a tumor [1], which is an important factor in treatment resistance. Pre-treatment DCE-MRI, therefore, may be able to predict which primary and nodal SCC sites in the head and neck will fail treatment. This information would be valuable not only for the radiation oncologist, to target specific SCC sites for a radiotherapy boost, but also for the radiologist to identify SCC sites with a higher likelihood of residual cancer, so that these sites can undergo post-treatment biopsy or close follow-up by imaging. In addition post-treatment DCE-MRI of a residual mass in the primary or nodal bed may improve the diagnostic ability of MRI to distinguish a residual mass that still contains cancer from one that is benign, so that primary or nodal resection can be performed before the cancer has chance to regrow and render the patient inoperable.

DCE-MRI shows promise for predicting and monitoring treatment response for a range of cancers outside of the head and neck [2, 3]. In regard to head and neck SCC, several DCE studies have shown favorable outcomes for SCCs with high vascularity on the pre-treatment DCE-MRI [4–13], but DCE methods, parameters and thresholds are variable, and few studies correlate DCE parameters at a specific site with treatment response at the same site. There are even fewer post-treatment DCE studies. Post-treatment DCE-MRI has shown a favorable response in a residual mass when there is later or less enhancement [11, 14–15], but using pharmacokinetic models an increase in vascular markers on the post-treatment DCE-MRI has been associated with a favorable response [16].

The aim of this study was to evaluate the ability of DCE-MRI to identify SCC sites in the head and neck that are at risk of treatment failure using a pharmacokinetic model. We report a prospective study in which DCE-MRI of the head and neck was performed pre-treatment for prediction and post-treatment for diagnosis. The DCE-MRI data was obtained from individual SCC sites in the head and neck and compared with treatment response at the same site, based on clinical outcome at that site at a minimum of two years.

## Methods

### Patients

This prospective study was approved by The Joint Chinese University of Hong Kong ­ New Territories East Cluster Clinical Research Ethics Committee(The Joint CUHK-NTEC CREC) and conducted following informed written consent. Patients with head and neck SCC treated with curative intent with RT or CRT were included in the study. The pre-treatment diagnosis of SCC at the primary site was made on histology and at nodal sites on MRI imaging criteria {a minimum axial diameter of ≥10 mm with the exception of jugulodigastric (≥11mm) and retropharyngeal nodes (≥5mm), or any sized node with necrosis or extra nodal neoplastic spread}. Radiotherapy was delivered to the primary tumor, metastatic regional lymph nodes, and sites at risk of microscopic spread to 54 Gy/ 30 fractions/ 6 weeks. A concomitant boost (18 Gy/ 12 fractions) was given to the gross tumor as a second daily dose in the last 12 treatment days. Concurrent chemotherapy consisted of intravenous cisplatin 40 mg/m^2^ given weekly on days 1, 8, 15, and 22 of RT.

### DCE-MRI data acquisition

MRI was performed on a 3T MRI scanner (Achieva, Philips Healthcare, Best, The Netherlands) using a 16 channel sensitivity encoding head and neck coil. Before performing DCE-MRI, routine protocol T1 and T2 weighted images were acquired. The DCE-MRI sequence was obtained using a short 3D T1-weighted spoiled gradient echo sequence in the axial plane covering the entire tumor (TR = 4.0 ms, TE = 1.0ms, flip angle = 15°, matrix = 128 × 128, number of slices = 25, slice thickness = 4 mm, number of signal averages = 1, sensitivity encoding factor = 3, temporal resolution = 2.59s/dynamic, number of dynamics = 185, and scanning time = 460s). Before dynamic acquisition, a flip angle of 2° was used with other parameters identical to dynamic acquisition to derive the pre-contrast T1 map. Contrast injection was commenced 6 seconds after the start of the dynamic MRI acquisitions, given in the form of a bolus injection of gadopenetate dimeglumine (Dotarem, Guerbet, France) at a concentration of 0.1 mmol/kg of body weight. A power injection pump (Medrad, Pittsburgh, PA) was used set at an injection rate of 3.0 ml/s through a 21-gauge intravenous catheter in an antecubital vein. The injection was followed by a 20-ml saline flush at the same injection rate. Following the DCE-MRI scan, post-contrast enhanced anatomical T1-weighted images were acquired as part of the routine clinical examination. The post-treatment scan was performed using the same DCE-MRI protocol 6 weeks +/- 3 weeks following the end of treatment.

### DCE-MRI data processing and analysis

DCE-MRI data were exported in a Philips PAR/REC format and then processed using the Philips PRIDE research tool v5.2 (Philips Healthcare, Best, The Netherlands) with the DCE-MRI images non-rigidly registered to the first dynamic image to compensate for possible patient movement. Voxels for arterial input function extraction were manually selected from the carotid arteries. Time intensity curves for arteries and tumors were converted into time-concentration-curves using the dual-flip-angle (2° and 15°) derived pre-contrast T1 map [17], a contrast agent r1 relaxivity value of 4.5s^-1^mM^-1^ and an assumed hematocrit value of 0.42. The DCE-MRI pharmacokinetic parameter maps were generated using the extracted arterial input function based on the Tofts model [18, 19].

The entire volume of each pre-treatment SCC site (primary site or metastatic nodal site) and any residual masses at these SCC sites, were outlined on the T1W dynamic images by a specialist head and neck radiologist using the T2-weighted and T1-weighted post-contrast axial images for guidance. The voxel-wise K^trans^ min^-1^, (the volume transfer constant between the blood plasma and extracellular extravascular space), k_ep_ min^-1^, (rate constant between the extracellular extravascular space and blood plasma), v_e_ (volume of the extracellular extravascular space per unit volume of tissue) were calculated using the non-linear least-square fitting algorithm using Philips PRIDE software. The goodness-of-fit (R^2^) was set as 0.7 for fitting. The area under the gadolinium concentration-time curve (AUGC) was also calculated for the whole time-concentration-curve.

The volume of each pre-treatment SCC site and the volume of any residual post-treatment mass at that site were measured.

### Assessment of treatment response

DCE-MRI parameters of each SCC site (primary or nodal) were correlated with subsequent site control (SC) or site failure (SF) at the same site. SF was determined by histological confirmation of SCC; radiological evaluation (increase in the size of a residual mass in the tumor bed or development of a new mass in the tumor bed on follow-up imaging); clinical evaluation (visualization of a definite tumor at the primary site, or increase in size of a primary or nodal mass on follow-up). SC was defined by absence of SCC at surgical resection or absence of SF on follow-up for least two years after the end of treatment.

### Statistical Analysis

The mean K^trans^, k_ep_, v_e_, AUGC and volume were evaluated. Univariate logistic regression analyses of each parameter pre-treatment, post-treatment, and percentage (%) change in each of these parameters from pre-treatment to post-treatment, were carried out to determine if there was a correlation between the DCE-MRI and volume parameters and treatment outcome at the same SCC site. Clinical factors were also assessed {age, sex, site (primary vs. node), T stage (T1/2 vs. T3/4), N stage (N0/N1 vs.N2/N3) and treatment regimes (RT, CRT and Induction CRT)}. Odds ratios and their corresponding 95% CIs were calculated in univariate analysis, parameters with *p*-values of less than 0.05 were included in a multivariate analysis with stepwise method. Adjusted odd ratios and corresponding 95% CIs were performed with confounding factors of site, T stage, N stage, tumor volume and treatment, in the model. Receiver-operating characteristics analyses with the area under the curve were employed to identify the optimal thresholds of significant parameters by giving equal weighting to sensitivity and specificity. The sensitivity, specificity, accuracy, positive predictive value and negative predictive value of this threshold were calculated, and the significance was re-evaluated with the Fisher’s exact test. In addition the paired t-test was used to compare mean DCE-MRI parameters and volumes pre-treatment and post-treatment at all sites with a residual post-treatment mass. All statistical tests were two sided, and *p*-values of less than 0.05 were considered to indicate a statistically significant difference. The software SAS version 9.3 (SAS Institute Inc, Cory, NC, USA) was used for statistical analysis.

## Results

### Patients

Pre-treatment DCE-MRI was performed on 85 patients with head and neck SCC. Of these 85 patients, 36 were excluded from the study; in 26 patients the planned treatment was changed to surgery, 5 patients subsequently did not undergo or complete a course of CRT/RT, 3 patients died before SF or SC could be confirmed, in one patient the AIF could not be obtained and in another the images were degraded. The study therefore comprised 49 patients (42 males and 7 females, median age 60 years, age range 41–93 years) presenting with primary SCCs of the oropharynx = 19; larynx = 14; hypopharynx = 12; and oral cavity = 4 (T stages were T1 = 1; T2 = 18; T3 = 13; T4 = 17). Nodal metastases were present in 35/49 patients (N0 = 14, N1 = 3, N2 = 31, N3 = 1). Patients underwent treatment with intensity modulated radiotherapy (IMRT) {with (1) or without (5) induction chemotherapy}, or IMRT concurrent with cisplatin or cetuximab {with (10) or without (33) induction chemotherapy}. Following treatment, a post-treatment DCE-MRI could not be obtained for analysis in 6/49 patients; an MRI was not performed (n = 1), renal impairment prevented the administration of contrast (n = 2), or the MRI was delayed (n = 3). The remaining 43 patients underwent a post-treatment DCE-MRI (mean = 43.3 days, median = 44.0 days, range = 23–62 days).

Pre-treatment DCE data in the 49 patients were obtained from primary and/or nodal sites {primary site = 19; nodal site = 5; two nodal sites = 1; primary site + one nodal site = 18; primary site + > one nodal site = 6 (2 nodal sites = 4, 3 nodal sites = 1, 4 nodal sites = 1)}. SF occurred in 21/49 patients (primary site = 9; one nodal site = 5; primary site + one nodal site = 6; primary site + two nodal sites = 1). Of the 31 patients with a residual mass (primary site = 17; nodal site = 12; primary site + one nodal site = 2), SF occurred in 13/31 patients (primary site = 6; nodal site = 6; primary site + one nodal site = 1).

### SCC Sites

Pre-treatment 83 SCC sites underwent DCE, 43 primary sites and 40 nodal sites (minimum axial diameter of metastatic nodes ranged from 11mm-35.6mm; mean 16.9mm). SC occurred in 54/83 sites (primary site = 27; nodal site = 27) at a mean of 42.6 months, median 39.8; range 24–68.0 months of follow-up time for patients with conservative management without salvage surgery. SF occurred in 29/83 sites (primary site = 16; nodal site = 13) at a mean of 7.2 months; median 6.1 months, range 1.3–34.5 months.

Post-treatment 71/83 sites underwent DCE-MRI, of these 38 were excluded from analysis because there was no residual mass or the residual mass was too small or necrotic for analysis. Therefore, 33 sites with a residual mass underwent both pre-treatment and post-treatment DCE-MRI analysis, of which 19 had SC (primary site = 12; nodal site = 7) at a mean 43.1 months, median 41.2 months, range 24.0–68.0 months, and 14 had SF (primary site = 7; nodal site = 7) at a mean 6.0 months, median 6.2 months, range 1.6–14.26 months.

### DCE-MRI, Volume and Clinical parameters

#### 1. Pre-treatment parameters at SCC sites correlated with SF or SC

The mean K^trans^, k_ep_, v_e,_ AUGC, volume and clinical factors for all 83 SCC sites in the head and neck pre-treatment are shown in Table 1. The pre-treatment K^trans^, k_ep_, v_e,_ and AUGC were higher and volume lower in SCC sites with SC compared to SF, but the results were not statistically significant (Table 1). Clinical parameters were not significantly different between sites with SC and SF (Table 1). When the model was adjusted for confounding factors none of the DCE parameters were significant. When the primary and nodal sites were analyzed separately the DCE parameters and volumes of these sites showed no correlation with treatment outcome either (Table 2).

**Table 1:** Pre-treatment mean DCE, volumes and clinical parameters in 83 SCC sites (primary plus nodal) correlated with SF or SC at the same site, based on a minimum of 2 year clinical follow-up.

Parameter	Pre-treatment Parameters for all SCC sites	Pre-treatment SCC sites with Site Failure (SF)	Pre-treatment SCC sites with Site Control (SC)	*p*-value comparing SCC sites with SF & SC using Logistic Regression	Odds Ratio (OR)	95% CI for OR
	n = 83	n = 29	n = 54			
K^trans^ (min^-1^)	0.37 ± 0.19	0.33 ± 0.14	0.39 ± 0.21	0.212	0.195	0.015–2.544
k_ep_ (min^-1^)	0.67 ± 0.30	0.64 ± 0.25	0.68 ± 0.32	0.575	0.642	0.136–3.024
v_e_	0.56 ± 0.21	0.53 ± 0.17	0.58 ± 0.23	0.319	0.327	0.036–2.941
AUGC (mM s)	2.38 ± 0.79	2.15 ± 0.70	2.50 ± 0.81	0.063	0.555	0.299–1.032
Volume (cm^3^)	4.35 ± 5.63	5.19 ± 7.11	3.90 ± 4.65	0.327	1.040	0.961–1.126
Age	61.3 ± 8.8	62.4 ± 8.7	60.6 ± 8.9	0.385	1.023	0.972–1.078
Sex Male (compared to female)	74	27	47	0.404	2.011	0.390–10.378
T Stage T3/4 (compared to T1/2)	57	20	37	0.967	1.021	0.385–2.704
N Stage N2/3 (compared to N0/N1)	68	24	44	0.156	2.404	0.715–8.076
Site Primary site (compared to nodal site)	43	16	27	0.653	1.231	0.498–3.044
Treatment				0.174	0.546	0.229–1.305
1. RT	6	2	4			
2. CRT without induction CRT	56	23	33			
3. Induction chemo CRT	21	4	17			

± Standard deviation.

**Table 2:** Pre-treatment mean DCE parameters in 43 primary sites and 40 nodal sites correlated with SF or SC at the same SCC site, based on a minimum of 2 year clinical follow-up.

Parameter	Pre-treatment for all SCC sites	Pre-treatment SCC sites with Site Failure (SF)	Pre-treatment SCC sites with Site Control (SC)	*p*-value comparing SCC sites with SF & SC using Logistic Regression	Odds Ratio (OR)	95% CI for OR
**Primary Tumor Sites**	n = 43	n = 16	n = 27			
K^trans^ (min^-1^)	0.38 ± 0.18	0.37 ± 0.16	0.39 ± 0.19	0.731	0.539	0.016–18.243
k_ep_ (min^-1^)	0.69 ± 0.32	0.70 ± 0.30	0.69 ± 0.33	0.916	1.112	0.154–8.015
v_e_	0.57 ± 0.23	0.54 ± 0.20	0.59 ± 0.25	0.498	0.385	0.024–6.062
AUGC (mM s)	2.44 ± 0.75	2.18 ± 0.60	2.59 ± 0.79	0.086	0.438	0.171–1.123
**Nodal Tumor Sites**	n = 40	n = 13	n = 27			
K^trans^ (min^-1^)	0.35 ± 0.20	0.28 ± 0.09	0.38 ± 0.24	0.157	0.047	0.001–3.250
k_ep_ (min^-1^)	0.64 ± 0.29	0.27 ± 0.16	0.67 ± 0.33	0.301	0.251	0.018–3.445
v_e_	0.54 ± 0.19	0.51 ± 0.12	0.56 ± 0.22	0.416	0.220	0.006–8.464
AUGC (mM s)	2.31 ± 0.84	2.12 ± 0.82	2.40 ± 0.84	0.324	0.655	0.282–1.520

± Standard deviation.

#### 2. Comparison between Pre-treatment and Post-treatment parameters at SCC sites with a residual mass

Comparing mean DCE parameters and volumes at SCC sites pre and post-treatment, there was a significant decrease in k_ep_ (0.67 ± 0.28 vs. 0.42 ± 0.24, p < 0.001) and volume (5.25 ± 6.31 vs. 1.10 ± 1.83, p < 0.001); a significant increase in v_e_ (0.57 ± 0.19 vs. 0.79 ± 0.23, p = < 0.001); no significant change in K^trans^ (0.37 ± 0.16 vs. 0.30 ± 0.15, p = 0.092) or AUGC (2.42 ± 0.71 vs. 2.49 ± 1.05, p = 0.732) using the Paired-t-test.

#### 3. Comparison of Post-treatment residual masses with SC and SF

**Fig 1:**
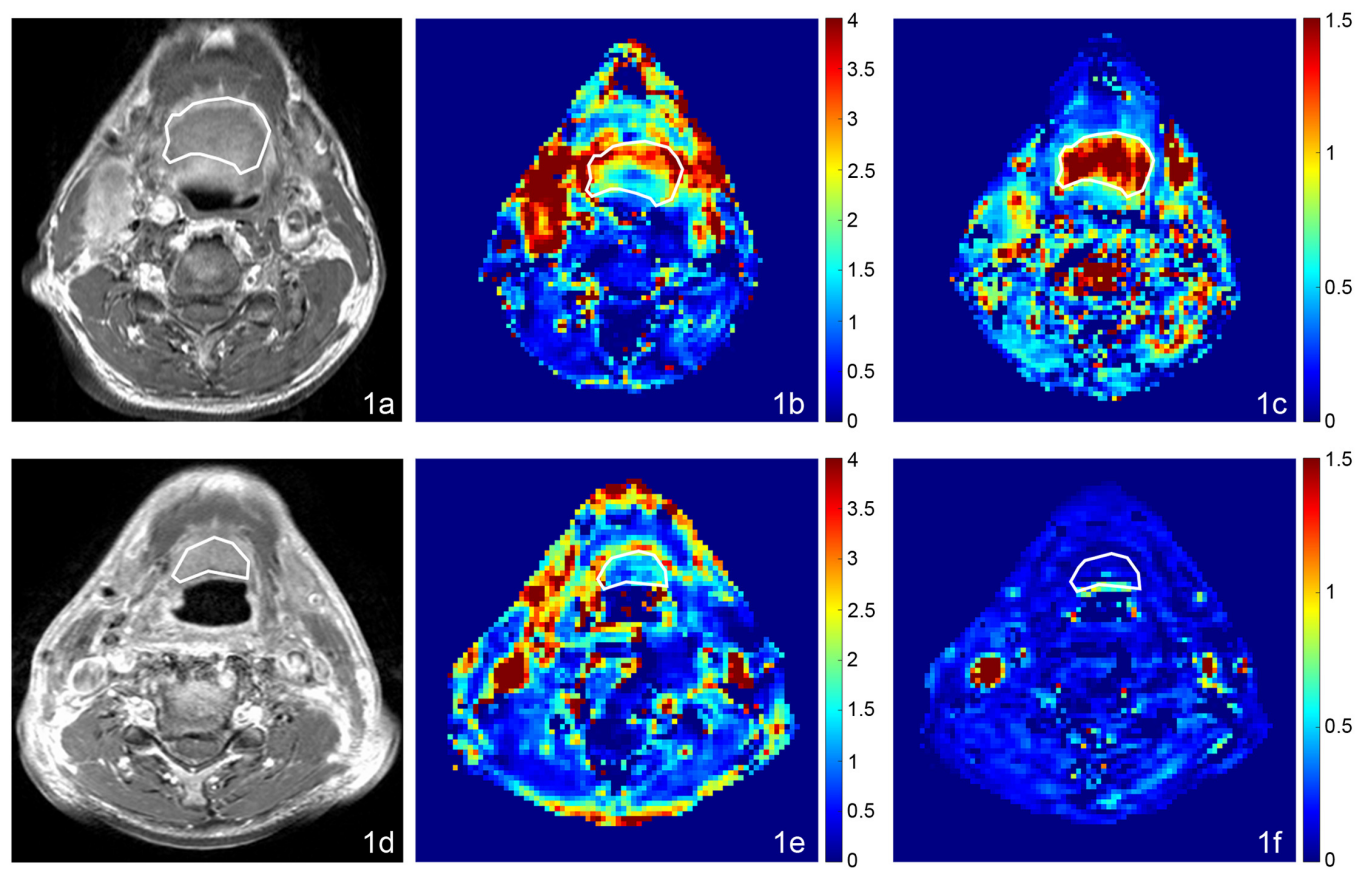
MRI, AUGC and k_ep_ maps in the axial plane from a 70 year old male with oropharyngeal carcinoma and SC 46.7 months after chemoradiotherapy. (a) Pre-treatment T1-weighted post contrast image of the SCC, (b) Pre-treatment AUGC map (mean AUGC = 2.36), (c) Pre-treatment k_ep_ map (mean k_ep_ = 1.62); (d) Post-treatment T1-weighted post contrast image of the SC residual mass, (e) Post-treatment AUGC map (mean AUGC = 0.96), (f) Post-treatment k_ep_ map (mean k_ep_ = 0.09).

**Fig 2:**
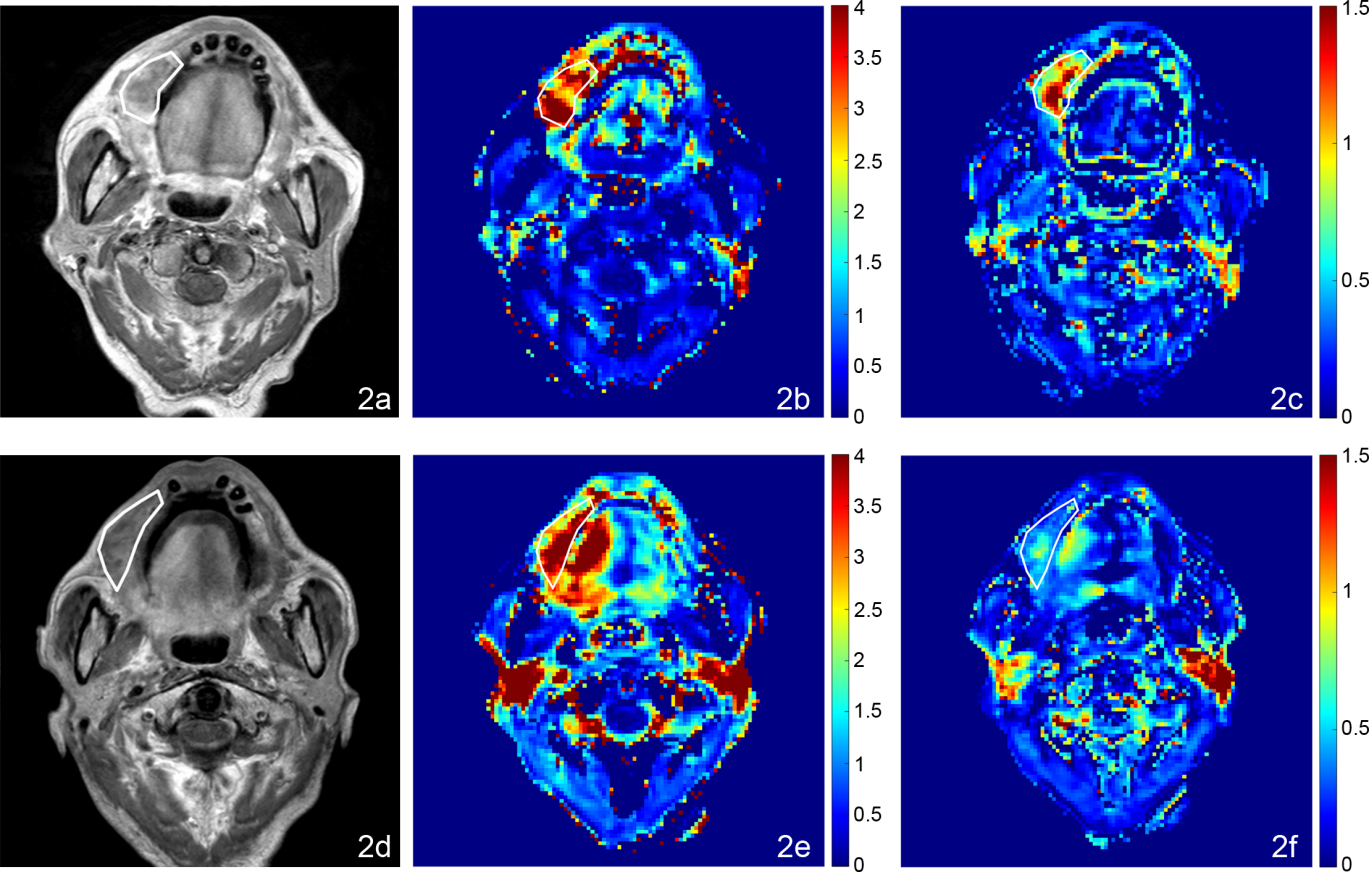
MRI, AUGC and k_ep_ maps in the axial plane from an 85 year old male with oral cavity carcinoma with SF 3.6 months after chemoradiotherapy. (a) Pre-treatment T1-weighted post contrast image of the SCC, (b) Pre-treatment AUGC map (mean AUGC = 3.07), (c) Pre-treatment k_ep_ map (mean k_ep_ = 0.88); (d) Post-treatment T1-weighted post contrast image of the residual mass with SF, (e) Post-treatment AUGC map AUGC (mean AUGC = 3.72), (f) Post-treatment k_ep_ map (mean k_ep_ = 0.63).

**Fig 3:**
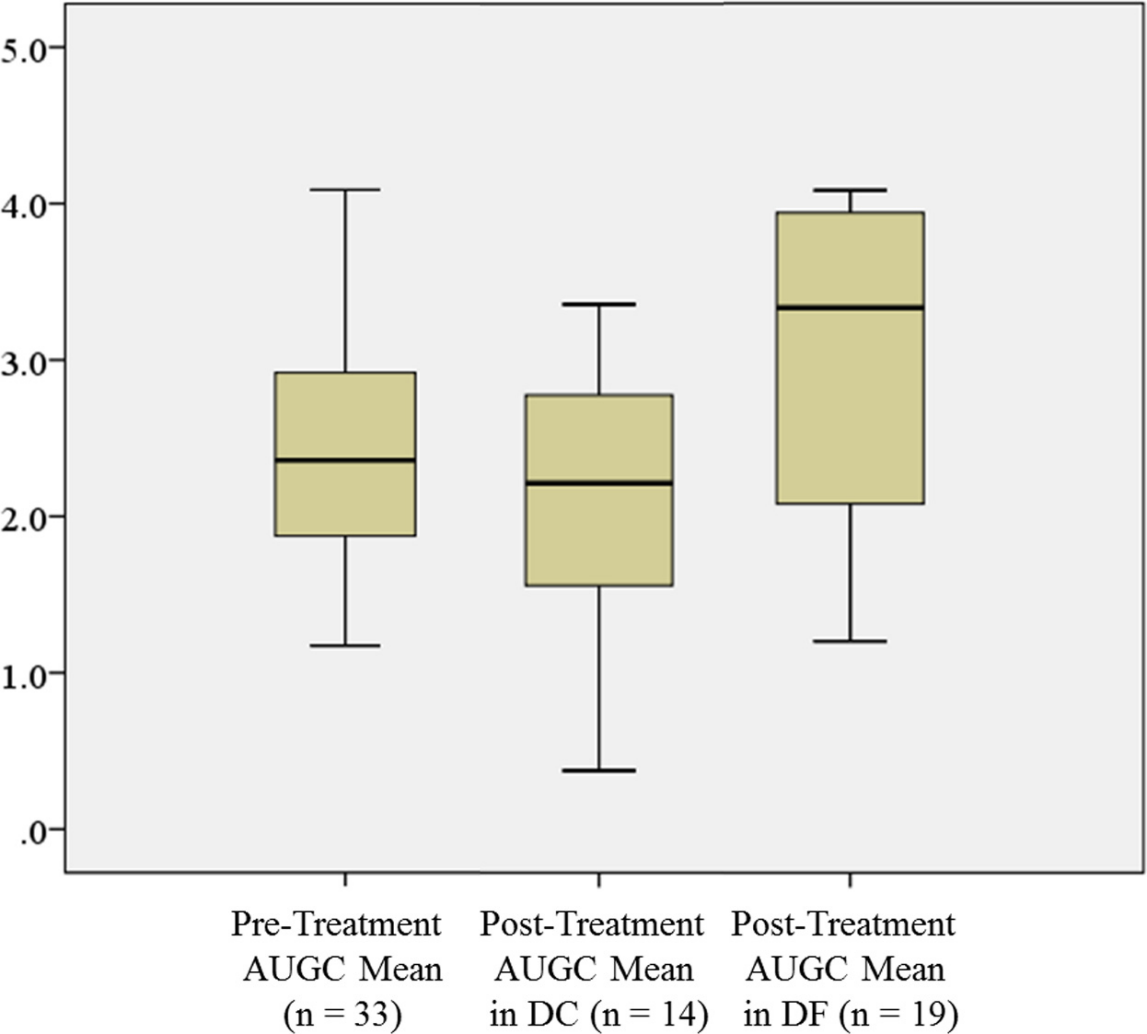
Box plots showing pre-treatment AUGC in 33 SCC sites and post-treatment AUGC in SC and SF residual masses. SC residual masses showed significantly lower AUGC than SF residual masses.

**Fig 4:**
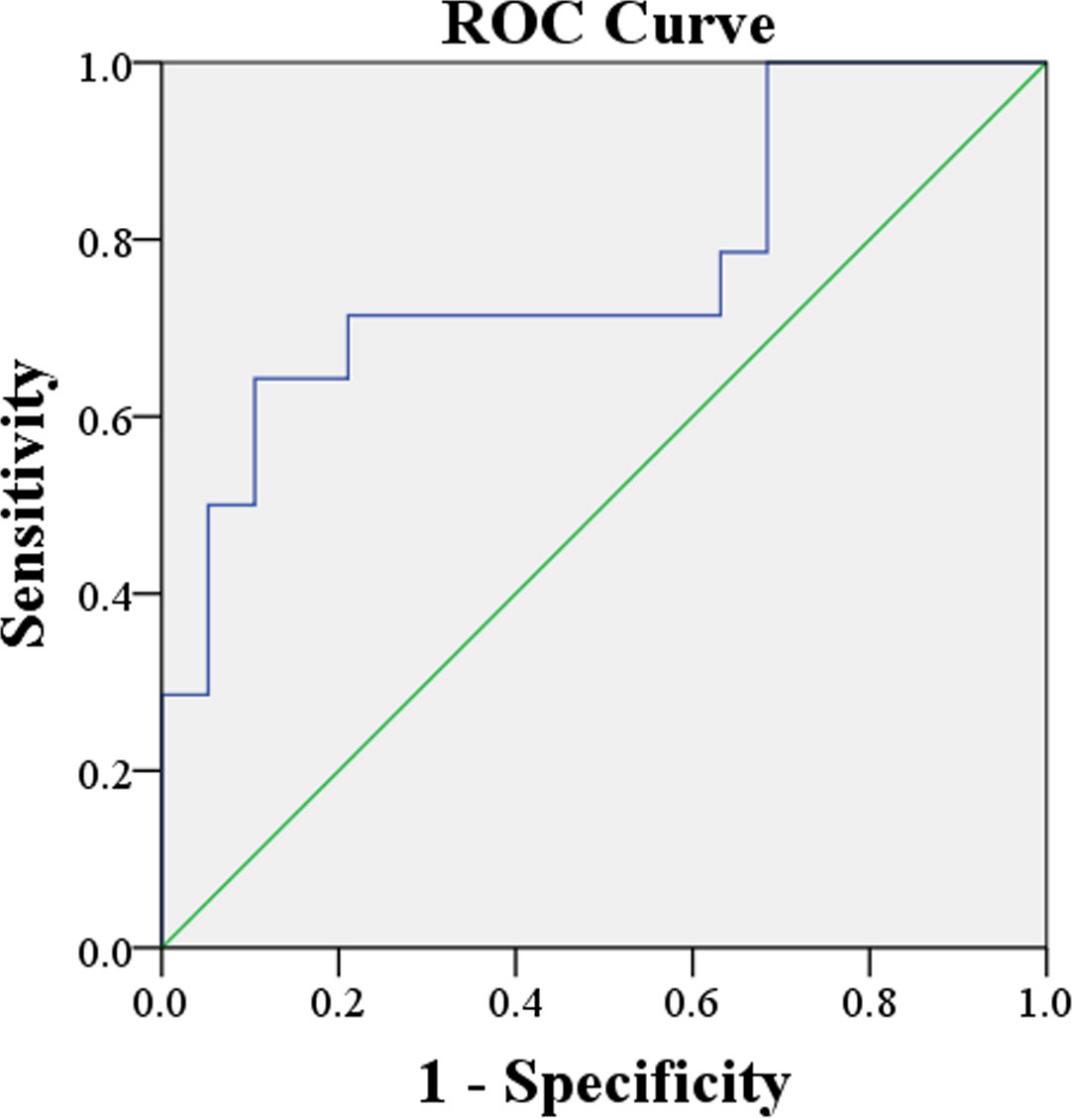
Receiver-operating characteristics curve. Receiver operating characteristic curve for the % change in AUGC of a post-treatment residual mass, for distinguishing between SC residual masses and SF residual masses. Area under the curve of 0.77 when using a > 19.78% increase in AUGC to predict SF.

The mean K^trans^, k_ep_, v_e_, AUGC and volume and respective % change compared to pre-treatment at 33 sites with a residual mass with SC and SF are shown in Table 3, together with the clinical parameters. SC residual masses showed significantly lower k_ep_ and AUGC compared with SF residual masses (p = 0.049 and p = 0.023 respectively) (Fig 1A–1F and Fig 2A–2F). In regard to % change when compared to pre-treatment SC residual masses showed a decrease in the AUGC while the SF residual masses showed an increase in AUGC (Fig 3), the % change in AUGC being significantly different between these two groups (p = 0.015). All other DCE parameters, volumes and clinical parameters showed no significant differences between residual masses with SC and SF. Multivariate analysis of significant post-treatment parameters (k_ep_, AUGC and the % change in AUGC) found the % change in AUGC remained significant (p = 0.015; OR 1.021; 95% CI for OR 1.004–1.039) and when the model was adjusted for the size and confounding factors, the % change in AUGC was still significant (p = 0.021; Adjusted OR 1.024; 95% CI for adjusted OR 1.003–1.044). Receiver-operating characteristics analysis (Fig 4) produced an area under the curve of 0.77 (95% CI = 0.594–0.940) and when using a > 19.78% increase in AUGC to predict a SF residual mass produced a diagnostic performance which is shown in Table 4. When the primary and nodal residual mass sites were analyzed separately the DCE parameters and volumes of these sites showed no correlation with treatment outcome (Table 5).

**Table 3:** Post treatment residual mass in 33 SCC sites (primary plus nodal): mean DCE and volumes (and % change when compared to pre-treatment), and clinical parameters for SC residual masses and SF residual masses, based on a minimum of 2 year clinical follow-up.

Parameter	Post-treatment Residual mass with Site Failure (SF)	Post-treatment Residual mass with Site Control (SC)	*p*-value comparing residual masses with SC & SF using Logistic regression	Odds Ratio (OR)	95% CI for OR
	n = 14	n = 19			
K^trans^ (min^-1^)	0.35 ± 0.14	0.26 ± 0.16	0.098	69.879	0.457–999.
% Change K^trans^	16.09 ± 71.80	-20.32 ± 50.93	0.129	1.012	0.997–1.027
k_ep_ (min^-1^)	0.52 ± 0.23	0.35 ± 0.23	**0.049**	29.692	1.017–866.979
% Change k_ep_	-15.90 ± 52.43	-34.27 ± 53.75	0.329	1.007	0.993–1.020
v_e_	0.75 ± 0.18	0.83 ± 0.26	0.353	0.232	0.011–5.072
% Change v_e_	53.10 ± 48.48	55.46 ± 90.80	0.928	1.000	0.990–1.009
AUGC (mM s)	3.00 ± 1.06	2.11 ± 0.89	**0.023**	2.639	1.146–6.073
% Change AUGC	42.64 ± 64.83	-12.63 ± 39.86	**0.015**	1.021	1.004–1.039
Volume (cm^3^)	1.47 ± 2.71	0.83 ± 0.66	0.399	1.264	0.733–2.180
% Change in volume	-0.68 ± 0.22	-0.74 ± 0.16	0.308	7.502	0.156–360.156
Age	63.8 ±10.7	61.8 ±11.2	0.585	1.018	0.954–1.087
Sex Male (compared to female)	13 (92.9)	14 (73.7)	0.186	4.642	0.477–45.197
T Stage T3/4 (compared to T1/2)	9 (64.3)	15 (79.0)	0.354	0.480	0.102–2.268
N Stage N2/3 (compared to N0/N1)	11 (78.6)	14 (73.7)	0.164	3.500	0.600–20.414
Site Primary site (compared to nodal site)	7 (50.0)	7 (36.8)	0.451	1.714	0.422–6.968
Treatment			0.115	0.179	0.021–1.515
1. RT	1	1			
2. CRT without induction CRT	13	13			
3. Induction chemo CRT	0	5			

± Standard deviation.

**Table 4:** Diagnostic performance of % change in AUGC to identify residual masses with SF.

Threshold	> 19.78
*p*-value	0.005
True positive	10
False positive	4
True negative	15
False negative	4
Sensitivity	71.4%
Specificity	78.9%
Accuracy	75.8%
Positive predictive value	71.4%
Negative predictive value	78.9%

**Table 5:** Post-treatment residual mass in 19 primary sites and 14 nodal sites; mean DCE parameters (and % change when compared to pre-treatment) for SC residual masses and SF residual masses, based on a minimum of 2 year clinical follow-up.

Parameter	Post-treatment Residual mass with Site Failure (SF)	Post-treatment Residual mass with Site Control (SC)	*p*-value comparing residual masses with SC & SF using Logistic regression	Odds Ratio (OR)	95% CI for OR
**Primary Tumor Sites**	n = 7	n = 12			
K^trans^ (min^-1^)	0.40 ± 0.15	0.30 ± 0.18	0.202	53.762	0.118–999.
% Change K^trans^	39.80 ± 95.63	-16.42 ± 58.81	0.167	1.011	0.995–1.028
k_ep_ (min^-1^)	0.50 ± 0.21	0.41 ± 0.26	0.422	5.324	0.090–314.83
% Change k_ep_	-17.09 ± 59.01	-19.63 ± 61.39	0.926	1.001	0.985–1.017
v_e_	0.76 ± 0.16	0.81 ± 0.25	0.635	0.341	0.004–28.829
% Change v_e_	63.36 ± 38.52	26.53 ± 52.66	0.132	1.018	0.995–1.041
AUGC (mM s)	2.96 ± 1.17	2.28 ± 0.77	0.147	2.326	0.744–7.266
% Change AUGC	33.18 ± 56.00	-10.12 ± 34.54	0.077	1.025	0.997–1.053
**Nodal Tumor Sites**	n = 7	n = 7			
K^trans^ (min^-1^)	0.31 ± 0.11	0.20 ± 0.10	0.136	-	-
% Change K^trans^	-7.62 ± 26.71	-26.98 ± 36.86	0.266	1.021	0.984–1.060
k_ep_ (min^-1^)	0.54 ± 0.26	0.23 ± 0.08	0.110	-	-
% Change k_ep_	-14.71 ± 49.71	-59.37 ± 24.34	0.133	1.050	0.985–1.118
v_e_	0.74 ± 0.22	0.85 ± 0.30	0.408	0.150	0.002–13.428
% Change v_e_	42.84 ± 57.99	105.05 ± 122.92	0.248	0.992	0.978–1.006
AUGC (mM s)	3.04 ± 1.03	1.83 ± 1.05	0.078	3.280	0.877–12.269
% Change AUGC	52.09 ± 75.90	-16.92 ± 50.90	0.093	1.019	0.997–1.042

± Standard deviation.

## Discussion

In this study none of the pre-treatment DCE-MRI parameters obtained from SCC sites in the head and neck were predictors of treatment response at the same SCC site based on clinical outcome at a minimum of two years. Although SCC sites with control showed higher pre-treatment perfusivity and permeability (K^trans^, k_ep_ and AUGC) and higher extracellular extravascular space (v_e_) compared to SCC sites with failure, none of these parameters proved to be significantly different between these two groups. On the other hand, treatment led to significantly lower tumor perfusivity (k_ep_) and higher extracellular extravascular volume fraction (v_e_), and after treatment the perfusivity of a residual mass with SC was significantly lower than that of a residual mass with SF, based on the k_ep_ and AUGC results. The % change in the AUGC, from pre-treatment to post-treatment was also a predictor of response, SCCs with less reduction in the AUGC were more likely to have SF. Interestingly the mean % change in the SF group showed an AUGC increase, whereas for the SC group there was an AUGC decrease. This suggests an increase in the AUGC after treatment could be a marker for residual head and neck SCC. However, the optimum threshold for the % AUGC change (>19.78% increase for SF) produced a relatively low diagnostic performance for DCE-MRI, especially when compared to the current imaging standard of ^18^FDG- PET/ CT at 3 months [20]. Of note the negative predictive value, which is valuable for excluding disease, was only 79% for DCE-MRI, compared to around 95% for ^18^FDG-PET/ CT [20].

Previous pre-treatment head and neck SCC DCE-MRI studies using pharmacokinetic models have found high mean K^trans^ [5, 7, 9,10], low K^trans^ skewness [6] or high v_e_ [9] to be predictors of a favorable outcome, based on prolonged disease free survival [5], progression free and overall survival in stage IV disease [6], non-response in nodes based on locoregional recurrence or death [7], neck control rates [9], and recurrence (primary, nodal or distant sites) [10]. However, the focus of our research was to evaluate the ability of DCE-MRI to predict treatment response at specific primary or nodal SCC sites in the head and neck, so that this information could be used to identify those sites at risk of failure after treatment. This would enable patients to undergo a post-treatment biopsy targeted to a specific SCC site or undergo ^18^FDG-PET/CT at 3 months. Those patients without a post-treatment diagnosis of residual cancer, who nevertheless had an abnormal DCE examination, could be selected for closer MRI follow-up with the aim of identifying residual cancers before they have chance to regrow and present later on as tumor recurrence. To the best of our knowledge only three pre-treatment studies have addressed this specific area using pharmacokinetic models [4, 8, 16]. Two of these studies found a correlation between high pre-treatment K^trans^ and SC, the first in 33 metastatic nodal SCC sites based on follow-up at 6 months [4] and the second in 58 primary SCC sites with follow-up period to death or a minimum of 12 months [8]. The third study based on histology from the surgical resection after CRT found no correlation between DCE-MRI parameters and treatment response [16] which is similar to our results. Therefore, at present there is insufficient data to determine whether pre-treatment DCE-MRI using pharmacokinetic modelling can be used to predict which SCC sites are at risk of treatment failure.

Post-treatment examination of residual abnormalities is an even greater challenge for DCE-MRI than pre-treatment examination of the tumor. To the best of our knowledge only one post-treatment SCC study used a pharmacokinetic model to evaluate similar DCE parameters to those in our study [16]. That study performed DCE-MRI two weeks after CRT and correlated the results with histology from the surgical specimen of 35 primary oral SCCs. A significant increase in v_e_, AUGC, and K^trans^, together with a significantly higher post-treatment v_e_ and AUGC were found in responders compared to non responders. Our results also showed an increase in the extravascular extracellular space (v_e_) in responders, (not significant), but our other DCE parameters had decreased after treatment. However, DCE-MRI data from time intensity curves in head and neck SCC [11, 15], and from pharmacokinetic models in breast and colorectal cancers, using k_ep_ and K^trans^ [21–25], are broadly in line with those from our study. We have observed a greater % reduction in the AUGC, and a lower AUGC and k_ep_ in SC residual masses compared to SF residual masses. The AUGC is considered to be more robust and simpler to quantify than those DCE-MRI parameters obtained from pharmacokinetic models, because it does not require an arterial input function and is less influenced by the complicated computation procedure in pharmacokinetic model fitting. The AUGC reflects the accumulation and/or depletion of contrast media within the cancer regardless of curve shape, although it has no direct correlation with the contrast circulation pathway, nor is it able to reflect any specific physiological processes such as perfusion or permeability. The AUGC is still subject to contrast injection protocol and dynamic imaging parameter setting and as such, cross-centre comparison of AUGC may become difficult. However, the % change in AUGC may be comparable across centers provided the DCE-MRI acquisition protocol is kept constant for the pre-treatment and post-treatment scans.

In this prospective study we used long-term clinical outcome with a minimum of 2 year follow up at each site, which is a superior to assessment of change in tumor size post-treatment. Nethertheless, there are several limitations to the study. We speculate that parameter quantification of pharmacokinetic modeling requires further development and could be one of the reasons for the insignificant correlation from the pre-treatment study. Parameter quantification of pharmacokinetic modeling is complicated and subject to many factors, including but not limited to tissue properties, contrast agent registration, DCE-MRI imaging protocol, as well as the fitting method [26]. One main concern is the accurate estimation of the arterial input function which is a problem encountered by all researchers in this field. Many methods have been proposed for obtaining the arterial input function, including the individual based arterial input function and population based arterial input function, but currently all methods have drawbacks [26]. In addition, several of our results obtained v_e_ values that were unexpectedly high, a problem also identified by other researchers including those studying head and neck SCC [4].

Head and neck SCC are heterogeneous tumors and so this study collected each DCE parameter from the entire tumor volume, rather than from small regions of interest or the largest cross sectional area. However, only the mean DCE parameter was measured and other measures for probing tumor heterogeneity were not assessed, such as assessment of the distribution of DCE parameters using skewness [6].

Seven patients in this study had data collection from more than one nodal site in the head and neck, one of whom had two sites of SF that were included in analysis of the pre-treatment results. DCE parameters and treatment response of individual nodes may vary even in a single patient and so we felt it was important to keep this data for site specific response, however this could lead to dependency issues in the statistical analysis. Not all patients had a residual mass in the tumor bed and even when a mass was present it may have been too small or too necrotic for DCE-MRI analysis. Therefore, although the sample size was sufficient to show significant differences between SC and SF for primary and nodal sites combined, the sample was not large enough to show a difference when primary and nodal sites were analyzed separately. Small residual masses in the primary tumor bed were particularly difficult to assess with DCE-MRI when they formed rims of tissue along the walls of the aerodigestive tract at the interface with air, as were necrotic nodes, so limiting the role of functional imaging in the post-treatment neck. Furthermore, contouring a residual abnormality in the post-treatment tumor bed can be more difficult than contouring the pre-treatment tumor because of the complex treatment induced signal abnormalities in the adjacent normal structures. Finally, patients with human papillomavirus (HPV) positive SCCs are more responsive to treatment with better treatment outcome, and successfully treated cystic nodal metastases may take longer to resolve [27, 28], but HPV status of many of the SCCs in this study were unknown and therefore the influence of HPV on treatment outcome for the oropharyngeal SCCs could not be assessed.

## Conclusion

In summary, there is little published data for head and neck SCC regarding the diagnostic ability of post-treatment DCE-MRI, using a pharmacokinetic model, to distinguish between a residual mass which is benign and one that still contains SCC. Compared to post-treatment residual masses with SF, those residual masses with SC showed significantly lower k_ep_ and AUGC and a greater % reduction in AUGC, the % change in AUGC remaining significant on multivariate analysis. In this respect the use of % change in AUGC is promising, but our results in the post-treatment are based on a relatively small patient population and further studies are needed. This study failed to identify any pre-treatment DCE-MRI parameters that were predictive of treatment failure at specific primary or nodal sites in the head and neck and DCE-MRI analysis using pharmacokinetic models remains a technically challenging technique to perform in the head and neck.

## Abbreviations

SCC: Squamous cell carcinoma
DCE-MRI: Dynamic contrast enhanced MRI
CRT: Chemoradiotherapy
RT: Radiotherapy
SC: Site control
SF: Site failure
K^trans^: Volume transfer constant between the blood plasma and extracellular extravascular space
k_ep_: Rate constant between the extracellular extravascular space and blood plasma
v_e_: Volume of the extracellular extravascular space per unit volume of tissue
AUGC: Area under Gadolinium concentration-time curve
